# Unveiling the Role of Nerve Conduction Studies (NCS) in Detecting Subclinical Peripheral Neuropathy in Autoimmune Disorders: A Cross-Sectional Study

**DOI:** 10.7759/cureus.70649

**Published:** 2024-10-01

**Authors:** Subbiah Senthilnathan, Gunasekaran Nallusamy, Priyadarshini Varadaraj, Keesari Sai Sandeep Reddy, Lokesh Kumar

**Affiliations:** 1 Internal Medicine, Saveetha Medical College and Hospital, Saveetha Institute of Medical and Technical Sciences, Saveetha University, Chennai, IND; 2 General Medicine, Adyar Cancer Institute, Chennai, IND

**Keywords:** early detection, incidence, positive association, risk factors in ctds, subclinical neuropathy

## Abstract

Introduction: Peripheral neuropathy, characterized by nerve damage, often presents with symptoms like pain, tingling, and muscle weakness. However, in its subclinical form, these symptoms may be subtle or absent, making early detection challenging. This is particularly concerning in patients with autoimmune connective tissue disorders (ACTDs), where the immune system attacks the body's tissues, potentially leading to nerve damage. Early identification and management are crucial to prevent the progression from subclinical to clinical neuropathy, which can significantly impair quality of life.

Materials and methods: This prospective cross-sectional study involved 100 patients with ACTDs of three or more years' duration, conducted at Saveetha Medical College over 15 months. Nerve conduction studies (NCS) were performed on bilateral ulnar, radial, sural, peroneal, and tibial nerves.

Results: Peripheral neuropathy was present in 18 (18%) of the study participants. Carpal tunnel syndrome was the most prevalent type, affecting 10 (55.56%) of those with neuropathy. Significant differences were found in the left peroneal motor nerve (p = 0.003) and right tibial nerve (p = 0.03) conduction times. Additionally, significant associations were observed between rheumatoid factor positivity (p = 0.011), anti-cyclic citrullinated peptide (anti-CCP) antibody status (p = 0.032), and the presence of peripheral neuropathy.

Conclusion: This study underscores the importance of early detection and intervention for peripheral neuropathy in patients with ACTDs. The study’s findings align with existing literature, suggesting that a substantial proportion of patients with ACTDs are at risk for peripheral neuropathy, particularly in older patients and those with specific autoimmune markers. Regular NCS assessments are recommended to identify at-risk individuals, potentially mitigating the progression of neuropathy and improving patient outcomes. Future research should include larger, more diverse populations and longitudinal studies to further validate these findings.

## Introduction

Peripheral neuropathy, a condition characterized by damage to the peripheral nerves, often manifests with symptoms such as pain, tingling, and muscle weakness. However, in its subclinical form, these symptoms may not be overtly apparent, making early detection challenging. Subclinical peripheral neuropathy can be particularly insidious in patients with autoimmune connective tissue disorders (ACTDs), where the immune system mistakenly attacks the body's own tissues, potentially leading to nerve damage. The early identification and management of peripheral neuropathy in these patients are crucial, as progression from subclinical to clinical neuropathy can significantly impair the quality of life and exacerbate the overall disease burden [1]. Nerve conduction studies (NCS) serve as a vital diagnostic tool in detecting peripheral neuropathy, especially in its subclinical stages. NCS measures the speed and strength of electrical signals as they pass through peripheral nerves, providing quantitative data that can identify early nerve dysfunction before clinical symptoms emerge [2]. Despite its potential, the role of NCS in the early detection of peripheral neuropathy in patients with ACTDs has not been thoroughly explored [3].

ACTDs, including conditions such as systemic lupus erythematosus, rheumatoid arthritis, and scleroderma, are known for their systemic effects, which often include the involvement of the peripheral nervous system. While clinical peripheral neuropathy is a recognized complication of these disorders, the subclinical form often remains underdiagnosed, leading to delayed intervention and poorer outcomes [4]. Given the chronic and progressive nature of ACTDs, early detection of nerve involvement through NCS could offer significant clinical benefits by enabling timely intervention [2,5].

Research has indicated that early intervention in subclinical neuropathy, particularly through prophylactic treatments, can mitigate the progression to overt peripheral neuropathy [5]. However, there is a paucity of prospective studies focusing on the utility of NCS in detecting subclinical neuropathy among patients with long-standing ACTDs. This gap highlights the need for a comprehensive study to explore the incidence of subclinical neuropathy and the potential for early intervention [6]. The rationale for this study stems from the need to improve the early detection and management of peripheral neuropathy in patients with ACTDs. By focusing on individuals who have lived with ACTDs for three or more years, this study aims to uncover the prevalence of subclinical neuropathy in this population. Utilizing NCS as a diagnostic tool, the study will identify those at increased risk of developing clinical neuropathy, thereby providing an opportunity for early prophylactic treatment.

The primary objective of this research is a clearer understanding of the incidence of subclinical neuropathy in ACTDs and the effectiveness of early interventions. Ultimately, this could lead to the development of standardized protocols for the early detection and management of peripheral neuropathy in patients with autoimmune disorders, improving long-term patient outcomes [5].

## Materials and methods

Study design and patient population

A prospective cross-sectional study was conducted on a total of 100 patients who had a consecutive duration of three or more years with ACTDs. The study was carried out at Saveetha Medical College over a period of 15 months, commencing from the date of obtaining ethical clearance. All patients with ACTDs for a consecutive period of three or more years at Saveetha Medical College and Hospital, who provided informed consent for participation in the research, were included. Patients with established peripheral neuropathy, diabetic patients, and those with hypothyroidism were excluded. Additionally, patients who have malignancy and those with psychiatric illnesses who were not cooperative for NCS were excluded.

Data collection and outcome measures

An NCS (Electrophysiological Evaluation) was conducted to confirm mononeuropathy. The proximal compound muscle action potential (CMAP) was used for accurate assessment. Latency and amplitude in a unilateral lesion were compared to the normal side. A small pulse of electrical current was used to stimulate the motor, sensory, or mixed nerves through the skin. Recording electrodes were placed on the skin over the nerves, and the electrical responses generated by the stimulation were recorded. The quantifiable parameters included response amplitude, response morphology, and velocity or latency of conduction along the stimulated path. Late responses, such as the F wave and the H reflex, were also measured, which inferred the integrity of a nerve's proximal portions and corresponding nerve roots.

Demyelinating diseases were distinguished by observing slow conduction velocity, prolonged distal latencies, conduction blocks, dispersion of the motor response waveform, and prolonged late responses. Axonal pathology reduced the amplitude of responses. Acquired focal or segmental demyelination showed conduction block, evident as a decrease in motor response amplitude at the proximal region compared to the distally stimulated site. It was noted that NCS primarily examine axons with a large diameter, while small-diameter fibres in the peripheral nervous system's autonomic, temperature and pain-sensing portions generate electrical impulses that are too small to record.

An F wave is a late response that occurs at the interface between the peripheral nervous system and the central nervous system due to antidromic activation of motor neurons, involving conduction to and from the spinal cord. F waves were recorded from any distal muscle by stimulating the appropriate nerve. Supramaximal stimulation, which is above maximal, was employed. The cathode was placed proximal to the anode to avoid the anodal block. The recording electrode was positioned in a belly-tendon configuration, similar to motor nerve conduction (MNC) studies. The amplifier gain was adjusted to 20-500 µV per division, and the sweep speed was set to 5-10 ms per division. F waves were recorded from a relaxed muscle, and clearly definable F waves typically required responses with an amplitude greater than 20 µV. For clinical purposes, 10-20 F waves were considered adequate.

The MNC technique was performed on the medial and lateral plantar nerves. The recording electrode for the medial plantar nerve was placed on the belly of the abductor hallucis muscle, while for the lateral plantar nerve, it was placed on the abductor digiti quinti muscle. The reference electrode was placed 3 cm distal to the active electrode. Stimulation was applied below and above the medial malleolus. In the sensory nerve conduction technique, the recording electrode was placed just below the medial malleolus. The reference electrode was placed 3 cm distal to the active electrode. Stimulation was applied to the first toe for the medial plantar nerve and the fifth toe for the lateral plantar nerve. Sensory conduction was also performed antidromically by reversing the stimulating and recording electrodes. In the sural nerve conduction technique, the surface electrode was placed between the lateral malleolus and the Achilles tendon. The nerve was stimulated antidromically 10-16 cm proximal to the recording electrode, distal to the lower border of the gastrocnemius at the junction of the middle and lower third of the leg. The leg was relaxed and positioned laterally for convenience during recording.

The ulnar nerve, which runs superficially along its course, was tested using the ulnar nerve conduction technique. MNC of each segment was measured by stimulating Erb’s point, the axilla, elbow, wrist, and palm. CMAPs were recorded from forearm muscles, such as the flexor carpi ulnaris or flexor digitorum profundus. The active and reference electrodes were placed on the medial side of the fifth digit, with the active electrode on the abductor digiti minimi muscle (over the belly of the muscle) and the reference electrode 3 cm distal to the active electrode or at the first interphalangeal joint. A ground electrode was placed on the palm. Stimulation was applied to the nerve supplying the muscle, with the cathode closest to the recording electrode. Supramaximal stimulation was given. The ulnar nerve was stimulated at the wrist, elbow, axilla, and Erb’s point. At the wrist, stimulation was given medially adjacent to the flexor carpi ulnaris tendon. Below and above the elbow, stimulation was applied 3.4 cm distal to the medial epicondyle and 10 cm distal to the medial epicondyle above the elbow.

Bilateral MNC studies, including F waves, were performed on the median, ulnar, peroneal, and posterior tibial nerves. Bilateral median, ulnar, superficial peroneal, and sural sensory NCS were performed. The belly tendon method was used for motor NCS, and sensory nerve conduction was studied in an antidromic fashion.

Statistical analysis

The data from the NCS were analyzed using both descriptive and inferential statistics. Descriptive statistics, such as means, medians, standard deviations, and frequencies, summarized the demographic and clinical characteristics of the patients, as well as the NCS results, including latency, amplitude, and conduction velocity for each tested nerve. Inferential statistics were employed to identify significant differences and associations; a t-test was used to compare nerve conduction parameters between patients with and without subclinical peripheral neuropathy, while a Chi-square test assessed the association between different autoimmune disorders and the presence of subclinical neuropathy. Logistic regression analysis identified independent predictors of subclinical neuropathy, considering variables like age, gender, duration of disease, and type of autoimmune disorder. A p-value of less than 0.05 was considered statistically significant, and the analysis was conducted using the Statistical Package for the Social Sciences (IBM SPSS Statistics for Windows, IBM Corp., Version 26.0, Armonk, NY).

## Results

**Figure 1:**
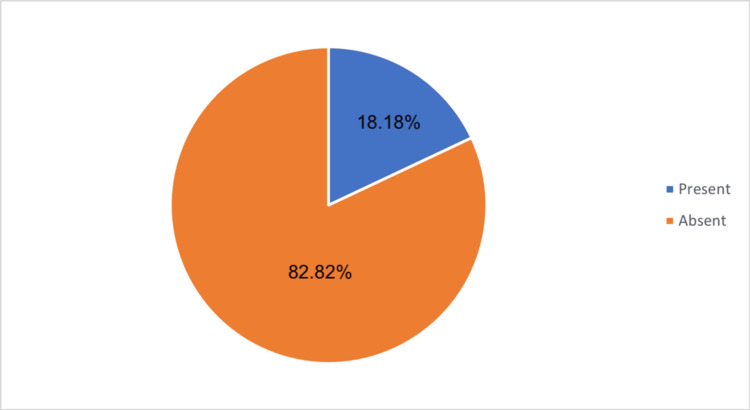
Incidence of peripheral neuropathy among study participants

Figure 1 illustrates the incidence of peripheral neuropathy among the study participants. According to the data, 18 (18%) of the patients were found to have peripheral neuropathy, while the remaining 82 (82%) did not exhibit signs of the condition.

**Figure 2:**
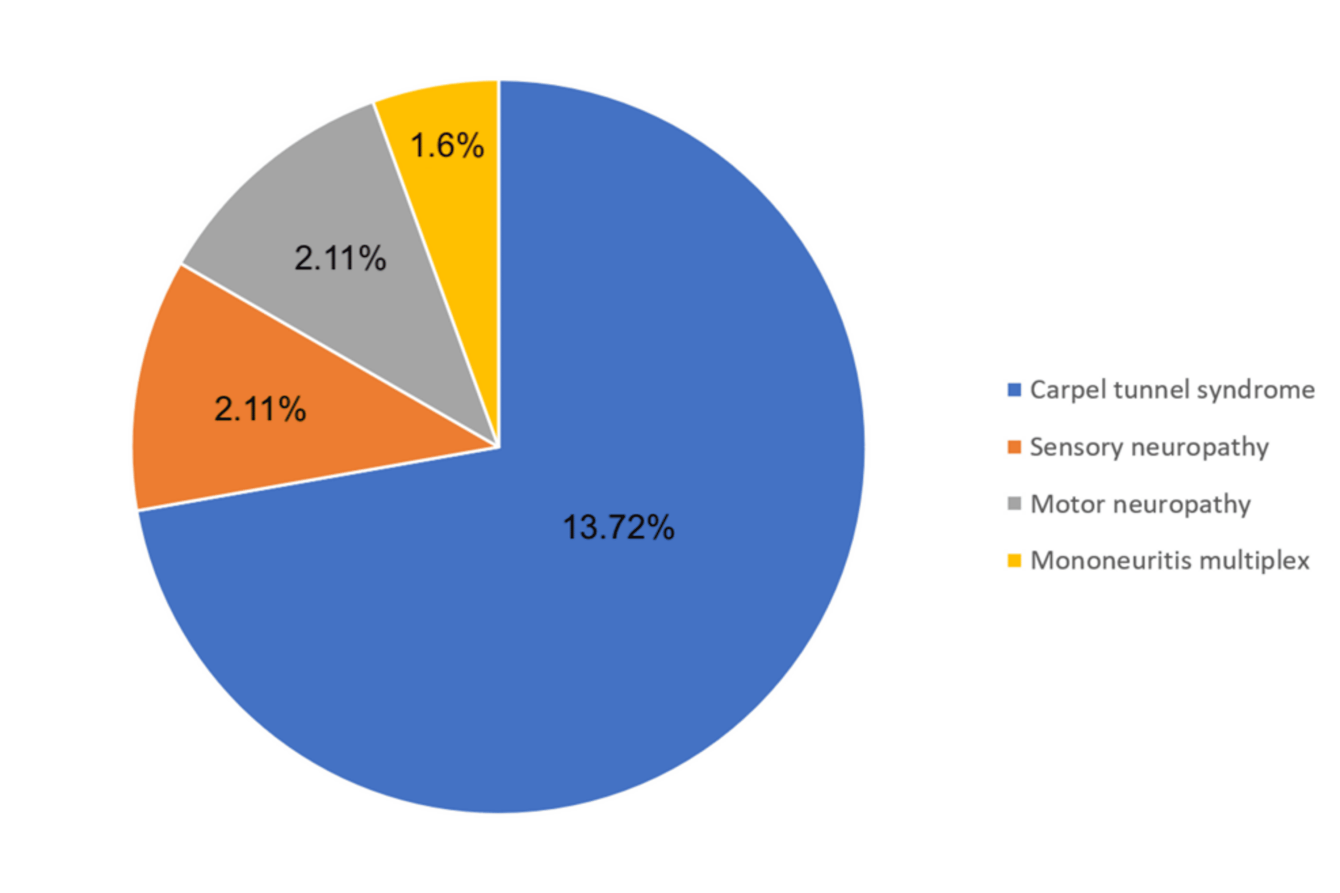
Distribution of various types of neuropathy among group 1 participants

Figure 2 displays the distribution of different types of neuropathy among the study participants diagnosed with ACTDs. The most prevalent condition was carpal tunnel syndrome (CTS), which affected 13 (72%) of group 1 participants, indicating it as the dominant form of neuropathy within this group. Sensory neuropathy was observed in two (11%) of group 1 participants. Motor neuropathy was observed in two (11%) of group 1 participants, highlighting that these types of neuropathy also contribute significantly, though to a lesser extent. Mononeuritis multiplex was the least common, affecting only one (6%) of the group 1 participants.

Table 1 presents that the baseline characteristics show a statistically significant difference in age between the two groups, with group 1 (peripheral neuropathy +ve) having a higher mean age (49.66 ± 4.77) compared to group 2 (peripheral neuropathy -ve) (46.02 ± 12.7), with a p-value of 0.047. Gender distribution and duration of ACTD did not show significant differences between the groups.

**Table 1:** Comparison of baseline characteristics between the two groups ACTD: autoimmune connective tissue disorder

Parameter	Group 1 (Peripheral Neuropathy +ve) (n=18)	Group 2 (Peripheral Neuropathy -ve) (n=82)	p-value
Age (mean ± SD)	49.66 ± 4.77	46.02 ± 12.7	0.047
Male N (%)	05 (27.8%)	17 (20.7%)	0.513
Female N (%)	13 (72.2%)	65 (79.3%)	
Duration of ACTD (years) N (%)			
≤5	05 (27.8%)	31 (37.8%)	0.478
6-10	07 (38.9%)	34 (41.5%)	
11-15	04 (22.2%)	11 (13.4%)	
16-20	0 (0%)	03 (3.7%)	
>20	02 (11.1%)	03 (3.7%)	

Table 2 shows that no significant differences were observed in the cytological parameters between the two groups. Haemoglobin levels, total leukocyte count, and other blood parameters were similar across both groups, with all p-values being greater than 0.05.

**Table 2:** Comparison of cytological parameters between the two groups ESR: erythrocyte sedimentation rate

Parameter	Group 1 (Peripheral Neuropathy +ve) (n=18)	Group 2 (Peripheral Neuropathy -ve) (n=82)	p-value
Haemoglobin (mean ± SD)	11.48 ± 1.75	12.08 ± 2.7	0.880
Total Leucocyte Count	9133.3 ± 2377.1	10221.9 ± 2403.9	0.084
Polymorphs (mean ± SD)	61.77 ± 4.18	63.17 ± 3.73	0.164
Lymphocyte (mean ± SD)	29.11 ± 5.23	28.70 ± 3.80	0.705
Monocyte (mean ± SD)	3.13 ± 0.514	2.99 ± 0.622	0.381
Platelets (lakhs/cumm) (mean ± SD)	2.65 ± 0.47	2.65 ± 0.483	0.923
ESR (mean ± SD)	33.22 ± 14.04	31.26 ± 12.52	0.559

Table 3 shows that the study found a significant difference in rheumatoid factor (RF) status (p = 0.011) and anti-cyclic citrullinated peptide (anti-CCP) antibody status (p = 0.032) between the two groups, indicating a potential link between these markers and the presence of peripheral neuropathy. However, C-reactive protein (CRP) status did not differ significantly between the groups (p = 0.841).

**Table 3:** Comparison of autoimmune inflammatory markers between the two groups Anti-CCP: anti-cyclic citrullinated peptide

Autoimmune Inflammatory Markers	Group 1 (Peripheral Neuropathy +ve) N (%)	Group 2 (Peripheral Neuropathy -ve) N (%)	Total N (%)	p-value
C-Reactive Protein (CRP) Status				
Positive	11 (61.1%)	48 (58.5%)	59 (59%)	0.841
Negative	07 (38.9%)	34 (41.5%)	41 (41%)	
Rheumatoid Factor Status				
Positive	08 (66.7%)	71 (58.5%)	79 (79%)	0.011
Negative	04 (33.3%)	11 (13.4%)	15 (15%)	
Anti-CCP Antibody Status				
Positive	11 (78.5%)	32 (47%)	43 (52.5%)	0.032
Negative	03 (21.5%)	36 (53%)	39 (47.5%)	

Table 4 shows that steroid medication usage was not significantly different between the two groups (p = 0.769), suggesting that steroid use did not have a major impact on the presence of peripheral neuropathy in the study population.

**Table 4:** Comparison of steroid use between the two groups

Steroid Medication Status	Group 1 (Peripheral Neuropathy +ve) N (%)	Group 2 (Peripheral Neuropathy -ve) N (%)	p-value
Yes	08 (16%)	42 (84%)	0.769
No	10 (20%)	40 (80%)	

Table 5 shows that significant differences were observed in the left peroneal motor nerve (p = 0.003) and right tibial nerve (p = 0.03) conduction times between the groups, suggesting that these nerves are more affected in patients with peripheral neuropathy. Other motor nerves did not show significant differences.

**Table 5:** Comparison of motor nerve function between the two groups

Motor Nerve (ms)	Group 1 (Peripheral Neuropathy +ve) Mean ± SD	Group 2 (Peripheral Neuropathy -ve) Mean ± SD	p-value
Left Median Motor Nerve	3.01 ± 0.49	3.21 ± 0.49	0.127
Right Median Motor Nerve	2.77 ± 0.40	2.74 ± 0.41	0.799
Left Ulnar Motor Nerve	2.38 ± 0.522	2.34 ± 0.422	0.712
Right Ulnar Motor Nerve	2.02 ± 0.58	1.85 ± 0.434	0.179
Left Peroneal Motor Nerve	3.94 ± 0.596	4.39 ± 0.559	0.003
Right Peroneal Motor Nerve	3.62 ± 0.41	3.81 ± 0.557	0.171
Left Tibial Nerve	4.22 ± 5.98	4.34 ± 0.60	0.465
Right Tibial Nerve	4.09 ± 0.844	3.69 ± 0.602	0.03

Table 6 shows that significant differences were found in the left ulnar sensory nerve (p = 0.05), right ulnar sensory nerve (p = 0.001), and right sural sensory nerve (p = 0.005) between the groups, indicating that these sensory nerves are particularly impacted in peripheral neuropathy. Other sensory nerves showed no significant differences.

**Table 6:** Comparison of sensory nerve function between the two groups

Sensory Nerve (ms)	Group 1 (Peripheral Neuropathy +ve) Mean ± SD	Group 2 (Peripheral Neuropathy -ve) Mean ± SD	p-value
Left Median Sensory Nerve	2.78 ± 0.37	2.86 ± 0.365	0.393
Right Median Sensory Nerve	2.47 ± 0.216	2.48 ± 0.32	0.915
Left Ulnar Sensory Nerve	2.43 ± 0.330	2.25 ± 0.399	0.05
Right Ulnar Sensory Nerve	2.13 ± 0.399	1.75 ± 0.397	0.001
Left Sural Sensory Nerve	2.18 ± 0.64	2.72 ± 0.690	0.583
Right Sural Sensory Nerve	2.21 ± 1.05	1.65 ± 0.596	0.005

Table 7 shows that the F-latency results show significant differences in the left median nerve (p = 0.05), right median nerve (p = 0.006), left peroneal motor nerve (p = 0.025), and right peroneal motor nerve (p = 0.001), indicating prolonged F-latency in these nerves among patients with peripheral neuropathy. Other nerves did not show significant differences.

**Table 7:** Comparison of F-latency between the two groups

F-Latency Nerve (ms)	Group 1 (Peripheral Neuropathy +ve) Mean ± SD	Group 2 (Peripheral Neuropathy -ve) Mean ± SD	p-value
Left Median Nerve	26.79 ± 1.89	27.77 ± 3.20	0.05
Right Median Nerve	27.15 ± 0.168	28.37 ± 1.65	0.006
Left Ulnar Nerve	26.92 ± 1.78	27.43 ± 1.48	0.205
Right Ulnar Nerve	27.60 ± 1.69	28.24 ± 1.59	0.135
Left Tibial Nerve	48.93 ± 2.94	48.39 ± 2.17	0.587
Right Tibial Nerve	48.09 ± 1.98	49.31 ± 2.19	0.542
Left Peroneal Motor Nerve	47.44 ± 2.179	48.60 ± 1.88	0.025
Right Peroneal Motor Nerve	47.39 ± 2.20	49.35 ± 1.942	0.001

## Discussion

The findings of this study emphasize the importance of early detection of subclinical peripheral neuropathy in patients with ACTDs, as the incidence of peripheral neuropathy was found to be 18% among the study participants. This incidence aligns with Bougea et al. [2], who reported that peripheral neuropathy affects 15-20% of patients with autoimmune conditions such as rheumatoid arthritis, systemic lupus erythematosus, and primary Sjögren’s syndrome. The 18% incidence observed in our study indicates a comparable prevalence, reinforcing the necessity for early diagnostic approaches, such as NCS, in the patient population.

Our study also revealed a significant association between RF and anti-CCP antibody positivity and the presence of peripheral neuropathy. In Group 1 (neuropathy-positive patients), 66.7% tested positive for RF, compared to 58.5% in Group 2 (neuropathy-negative patients) (p = 0.011). Additionally, 78.5% of neuropathy-positive patients were anti-CCP positive, compared to 47% of neuropathy-negative patients (p = 0.032). These findings align with those of Tulbă et al. [4], who found that patients with autoimmune conditions and positive RF or anti-CCP markers are more likely to develop nerve involvement, often preceding the onset of clinical symptoms. De Souza et al. [1] similarly noted that immunological markers like RF and anti-CCP are strong predictors of peripheral nerve damage in autoimmune diseases, particularly rheumatoid arthritis.

NCS results from our research revealed significant differences in motor and sensory nerve conduction times between neuropathy-positive and neuropathy-negative patients. The left peroneal motor nerve conduction time was notably slower in neuropathy-positive patients (3.94 ± 0.60 ms) compared to neuropathy-negative patients (4.39 ± 0.56 ms, p = 0.003), and the right tibial nerve conduction time was slower as well (4.09 ± 0.84 ms vs. 3.69 ± 0.60 ms, p = 0.03). These findings mirror those of Davalos et al. [3], who reported that slowed nerve conduction in vasculitic neuropathy patients indicates axonal damage and demyelination, both early signs of neuropathy. Sommer et al. [6] also highlighted that reduced motor nerve conduction velocities are characteristic of autoimmune-related peripheral neuropathies, supporting our study's observations of compromised motor nerve function in ACTD patients.

Our study also identified sensory and motor neuropathies, with sensory neuropathy affecting 11% of group 1 participants and motor neuropathy affecting 11% of group 1 participants, highlighting the broad spectrum of nerve involvement in ACTDs, as noted in the study done by Kaeley et al. [7]. Similarly, Young and Koduri [8] identified CTS as a predominant form of neuropathy in autoimmune disease patients, particularly those with long-standing rheumatoid arthritis.

The study by Sim et al. [9] also supports this observation, demonstrating that sensory nerve involvement is a key indicator of subclinical neuropathy in rheumatoid arthritis patients, as detected through NCS. CTS was the most common neuropathy identified in our study, affecting 55.56% of the neuropathy-positive participants. This finding is consistent with research by Borsook et al. [10], who observed a similar prevalence of CTS in patients with rheumatoid arthritis due to chronic inflammation and synovial hypertrophy, which leads to median nerve compression.

The sensory NCS revealed significant differences in the ulnar and sural nerves. For example, the right ulnar sensory nerve showed markedly slower conduction in neuropathy-positive patients (2.13 ± 0.40 ms) compared to neuropathy-negative patients (1.75 ± 0.40 ms, p = 0.001). The right sural nerve was similarly affected, with slower conduction in neuropathy-positive patients (2.21 ± 1.05 ms vs. 1.65 ± 0.60 ms, p = 0.005). These findings indicate that sensory nerves particularly those involved in temperature and pain sensation, are often affected in autoimmune neuropathies, even before motor nerve dysfunction becomes apparent.

The F-latency results in our study further support the presence of early subclinical neuropathy, particularly in the median and peroneal nerves. Prolonged F-latency in the left peroneal motor nerve was observed in neuropathy-positive patients (47.44 ± 2.18 ms) compared to neuropathy-negative patients (48.60 ± 1.88 ms, p = 0.025), with similar results for the right peroneal motor nerve (47.39 ± 2.20 ms vs. 49.35 ± 1.94 ms, p = 0.001). These findings align with research by Varshney et al. [11], who noted that F-latency prolongation is a sensitive marker of early nerve dysfunction, particularly in autoimmune conditions where motor nerves are frequently involved.

The association between age and the presence of peripheral neuropathy was another important finding in this study. Neuropathy-positive patients had a higher mean age (49.66 ± 4.77 years) compared to neuropathy-negative patients (46.02 ± 12.70 years, p = 0.047), which is consistent with the findings of Anwar et al. [5], who identified age as a significant risk factor for the development of peripheral neuropathy in patients with rheumatoid arthritis. This emphasizes the importance of regular neurological assessment in older patients with long-standing autoimmune diseases.

Peripheral neuropathy is managed through a variety of approaches depending on the underlying cause and severity of symptoms. Common treatments include medications such as anticonvulsants (e.g., gabapentin and pregabalin) and antidepressants (e.g., duloxetine), which have been shown to be effective in alleviating neuropathic pain by modulating nerve activity. Topical treatments, such as capsaicin cream and lidocaine patches, are also frequently used to reduce localized pain [12]. Physical therapy plays a crucial role in improving muscle strength and mobility, which can alleviate symptoms and improve quality of life.

The relatively small sample size, particularly in the group of patients with peripheral neuropathy, may limit the generalizability of the findings to the broader population with ACTDs. Conducting the study at a single centre introduces the possibility of selection bias, potentially limiting the applicability of the results to other clinical settings. Additionally, the cross-sectional design provides only a snapshot of the association between peripheral neuropathy and ACTDs, without allowing for the determination of causality or the progression of neuropathy over time. Longitudinal studies are needed to track the progression of peripheral neuropathy in ACTD patients over time, helping to understand the natural history of the condition and the impact of various treatments.

## Conclusions

This study aimed to uncover the incidence and characteristics of subclinical peripheral neuropathy in patients with ACTDs through NCS. The findings indicate that a significant proportion of patients with ACTDs may develop peripheral neuropathy, with specific motor and sensory nerves being particularly affected. The association of neuropathy with age, autoimmune markers such as rheumatoid factor and anti-CCP antibodies, underscores the importance of early detection and intervention. Despite the study's limitations, the results highlight the need for regular nerve conduction assessments in patients with ACTDs to identify those at risk for peripheral neuropathy. Early diagnosis and targeted treatment strategies could potentially mitigate the progression of neuropathy and improve patient outcomes. Future research should focus on longitudinal studies with larger, more diverse populations to further validate these findings and refine management approaches for peripheral neuropathy in ACTDs.
